# Are Men Who Buy Sex Different from Men Who Do Not?: Exploring Sex Life Characteristics Based on a Randomized Population Survey in Sweden

**DOI:** 10.1007/s10508-020-01843-3

**Published:** 2020-12-22

**Authors:** Charlotte Deogan, Elin Jacobsson, Louise Mannheimer, Charlotte Björkenstam

**Affiliations:** 1 The Public Health Agency of Sweden, SE-171 82 Solna, Stockholm, Sweden; 2 Karolinska Institutet, Department of Global Public Health Sciences, Social Medicine, Infectious Diseases and Migration Research, Stockholm, Sweden; 3 Karolinska Institutet, Department of Learning, Informatics, Management and Ethics, Stockholm, Sweden; 4 Department of Neuroscience, Psychiatry, Uppsala University, Uppsala, Sweden

**Keywords:** Sexual behavior, Sexual experience, Sexual health, Pornography, Sex work, Buying sex

## Abstract

The buying and selling of sex is a topic of frequent discussion and a relevant public health issue. Studies of sex workers are available, while studies addressing the demand side of sex are scarce, especially based on robust population data. The current study provides national estimates of the prevalence of and factors associated with having paid for sex among men in Sweden. We used a randomized population-based survey on sexual and reproductive health and rights among ages 16–84 years, linked to nationwide registers. The sample consisted of 6048 men. With a logistic regression, we analyzed what sex life factors were associated with ever having paid for or given other types of compensation for sex. A total of 9.5% of male respondents reported ever having paid for sex. An increased probability of having paid for sex was identified in men who were dissatisfied with their sex life (aOR: 1.72; 95% CI: 1.34–2.22), men reporting having had less sex than they would have liked to (aOR: 2.78; 95% CI: 2.12–3.66), men who had ever looked for or met sex partners online (aOR: 5.07; 95% CI: 3.97–6.46), as well as frequent pornography users (aOR: 3.02; 95% CI: 2.28–3.98) Associations remained statistically significant after adjustment for age, income, and educational attainment. Sex life characteristics such as poor sex life satisfaction, high online sex activity, and frequent pornography use are strongly associated with sex purchase. These findings can help guide and support counselling and prevention activities targeting sex buyers.

## Introduction

The buying and selling of sex is a topic of frequent discussion and a relevant public health issue. Transactional sex is generally defined as the trading (buying and selling) of sex for material benefit, i.e., exchanging money, drugs, food, shelter, or other items for sex (Carael, Slaymaker, Lyerla, & Sarkar, 2006; Stoebenau, Heise, Wamoyi, & Bobrova, 2016). The phenomenon has mainly been described as men paying women for sex, but increased attention has been paid to men and women paying men for sex as well (Berg, Molin, & Nanavati, 2020; Carael et al., 2006). While studies of sex workers and individuals receiving money or other types of compensation for sex are available and show considerable poor health (Halcón & Lifson, 2004; Miller et al., 2011; Seib, Fischer, & Najman, 2009; Ulloa, Salazar, & Monjaras, 2016; Wong, Holroyd, Gray, & Ling, 2006), studies that address the demand characteristics of sex based on robust population data are more scarce. Moreover, data providing sex life characteristics of sex buyers is unique in Scandinavia, and hence, the present study provides novel findings. In the UK, Ward et al. (2005) and Jones et al. (2015) provided estimates from nationally representative studies showing that 6–11% of British men had at some point paid for sex.

A 1996 survey including of 1145 Swedish men aged 18–74 years found that 12.7% of the respondents had paid for sexual services. (Månsson, 1996) Estimates from other Western and Northern European countries have shown that about 12.9% of Norwegian men (Schei & Stigum, 2010), 11–13% of Finnish men (Haavio-Mannila & Rotkirch, 2000) had at some point paid for sex. Paying or giving other types of compensation or reimbursement for sex is a crime in Sweden since 1999, when the purchase of sexual services became illegal. The law is aimed to increase gender equality and protect vulnerable women from exploitation and violence. The Swedish strategy for gender equality also includes the aim to reduce the demand for prostitution. A 2010 longitudinal internet survey among Swedes, Norwegians, and Danes aged 18–65 investigated the effects of criminalization on the demand and purchase of sex. In Norway, the purchase of sexual services is illegal since 2009, and in Denmark, it is still legal. The proportion who reported having bought sex during the past 6 months was lowest in Sweden (0.29%), higher in Denmark (1.3%) and in Norway (0.93%). The conclusion of the authors is that the effect of criminalization is a decrease in demand and purchase of sexual services (Kotsadam & Jakobsson, 2014). In the U.S., 16% of men reported having paid for sex at least once in their lives, and 0.5% reported doing so at least once a year (Michael, Gagnon, Laumann, & Kolata, 1994). In Russia, it was found that 10–13% of men had purchased sex at least once (Haavio-Mannila & Rotkirch, 2000). In Holland the comparable figure is 14%, in Switzerland 19%, in the UK 7–10%, and in Spain 39% (Leridon, van Zesson, & Hubert, 1998). Figures in the 70% range have been recorded for Cambodia and Thailand, but these, too, appear to be imprecise estimates (Ben-Israel & Levenkron, 2005; Della Giusta, Di Tommaso, Shima, & Strøm, 2009). A study highlights the prevalence of Swedish men paying for sex abroad, for example, in Thailand on vacation (Manieri, Svensson, & Stafström, 2013).

The underlying mechanisms and reasons for buying sex are complex and diverse. Additionally, to the physical act of sex, studies have described that reasons for buying sex varies across groups of men and also include, for example, emotions, need of intimacy, social connectedness, and wanting a relationship (Birch & Braun-Harvey, 2019; Monto & Milrod, 2014; Weitzer, 2007).

American research on men 60–84 years of age show advancing age to be positively associated with increased frequency of paying for sex. Those with higher incomes and without partners were more likely to report non-sexual activities with providers, and many participants sought a “girlfriend experience,” in which paid sexual exchanges are part of a relationship that mirrors conventional non-remunerative relationships (Milrod & Monto, 2017).

Studies comparing sex buyers to non-sex buyers have found that sex buyers are more likely, to report sexual aggression and likelihood to rape than men who do not pay for sex. Men who paid for sex scored higher on measures of impersonal sex and hostile masculinity and had less empathy for prostituted women (Farley, Golding, Matthews, Malamuth, & Jarrett, 2017). Findings from empirical studies of sex buyers suggest that background and personal characteristics are likely to affect the demand. These include self-perception, perceptions of women, sexual preferences, economic factors (education, income, work), as well as attitudes toward risk (health hazard and risk of being caught where sex work is illegal), lack of interest in conventional relationships, and desire for variety in sexual acts or sexual partners (Della Giusta, Di Tommaso, & Jewell, 2017).

A study performed by The Swedish National Council for Crime Prevention in 2008 showed that Swedish sex buyers are a heterogeneous group apart from the fact that the vast majority are men and not women (BRÅ, 2008). Buyers are from different socioeconomic backgrounds and of all ages, even though the most common ages are 30–50 years. Around 50% of buyers were highly educated and married. A population-based survey study by Priebe and Svedin (2011) showed Swedish buyers did not differ from non-buyers in terms of educational level or marital status. However, a range of other differences were identified among buyers: Higher proportion had experienced divorce or separation, higher number of change of partners, they were more often employed, while-non buyers were more often unemployed, students, retired or on sick leave, a higher proportion had high income, and a higher proportion had been travelling with work during the past year. Buyers, to a higher extent, had experience of violence in previous relationships, had experienced violence in childhood as well as experienced un-volitional sex. Alcohol and drug use were more common among buyers, and buyers had had more sexual partners and used the internet for sexual activity to a higher extent than non-buyers (Priebe & Svedin, 2011). Studies on selected groups of men having paid for sex suggest that these men are a high-risk group of sexually transmitted infections exposing both the sex workers and their other sexual partners. (Moore, 1999) Yet, knowledge of how sex life characteristics come into play in the demand for buying sex remains to be further explored.

### Aims

The aim of this study was to estimate the prevalence of and identify factors associated with having paid or given other types of compensation for sex among a randomized population-based sample of men in Sweden.

## Method

### Participants and Procedure

In the present study, we used data from SRHR2017 (sexual and reproductive health and rights), a randomized population-based survey including women and men between ages 16 and 84 in Sweden. The overall aim of the main research project, performed by the Public Health Agency of Sweden, was to explore a range of factors in sexual and reproductive health and rights.

Data collection was performed by Statistics Sweden, a governmental agency, during autumn of 2017. A randomized stratified sample of approximately 50,000 individuals aged 16–84 years of age were invited to participate in the survey either by answering online or paper–pencil by post. The sampling of participants was based on information from the Swedish Total Population register. This register was established in 1968 and includes information such as date of birth, age, sex, immigration dates, emigration dates, and place of residence. The sampling frame consisted of 7,906,368 individuals. A simple stratified random sample of 50,016 individuals was drawn. Due to overcoverage, 232 individuals were excluded, thus 49,784 remained and received the questionnaire. The survey questions were developed by the Public Health Agency of Sweden following an expert review carried out by Statistics Sweden. The final survey included 66 questions (118 including follow up questions).

The paper questionnaires were mailed and the respondents also received an information letter on the survey and its purpose. The respondents were also informed that the questionnaire would be supplemented with register data and that participation was voluntary. In total, three reminders were sent out. In total, 15,186 individuals responded, generating a response rate of 30.5%. Non-responders were more likely to be born outside of Sweden, to have lower educational level, to be men, and to be young. The partial non-response varied between 0 and 14% for the different questions. Another 639 respondents questionnaires were excluded due to contradictory responses, thus the sample consisted of 14,537 individuals. The results were weighted on basis of sex, age-group, region of residence, country of birth, and the highest attained educational level. Due to the weights, we can draw conclusions about the whole Swedish population, instead of just the individuals constituting the sample.

SRHR2017 was further enriched by linkage to the national Longitudinal Integration Database for Health Insurance and Labor Market Studies (LISA) From LISA, information on sex, age, country of birth, region of residence, immigration status, highest attained educational level and income was obtained for the respondents. Linking was possible due to the unique personal identity number addressed to all Swedish residents.

### Measures

The outcome variable having paid or given other type of compensation for sex was based on the question “Have you ever paid or given other compensation for sex”? Response alternatives included “yes, once,” “yes, several times,” “yes, the past year,” “yes, more than a year ago,” and “no.” The question was followed by an explanatory text “Other types of compensation can include clothes, presents, alcohol, drugs or a place to sleep, but also to obtain or advance to or keep a job.” The response alternatives were dichotomized and all alternatives of “yes” were categorized into “yes” and “no” into “no”. Respondents could check multiple boxes.

The following sociodemographic variables were included in the analyses: sex, age-group (16–29, 30–44, 45–64, 65–84), highest attained educational level (≤  9 years, 10–12 years and > 12 years), income level (5 groups: lowest income group (0–20) represent the 20% of individuals with lowest income, and the highest income group (80–100) represented by the 20% of individuals with highest income).

#### Variables of Sex Life

One question on sexual satisfaction and sexual dissatisfaction was asked, “What do you think about your sex life over the past 12 months?” Two response alternatives were provided: (1) I am mostly satisfied; (2) I am mostly dissatisfied. As the respondents could check both boxes, the 3604 individuals who did, were categorized into a third alternative interpreted as “both satisfied and dissatisfied.”

The question “What do you think about your sex life over the past 12 months?” was asked providing response alternatives “I lack a sex partner,” “I want more sexual partners,” “I have not had sex often enough,” and “I have not had sex in the way I would like to.” A new variable called “Having less sex than one would like to” was created by having answered “yes” on minimum two out of the four response alternatives.

A question was asked on sexual activities online: “Have you ever engaged in any of the following activities online, via mobile phone or via apps?” Response alternatives included: “looked for a sex partner” and “found a sex partner” (Yes/No). A new variable was created “having looked for or found a sex partner online” based on a “yes” answer on any of the two response alternatives.

Lastly, a question on pornography use was asked: “Do you watch pornography intentionally?” Response alternatives included: “Daily or almost daily,” “3–5 times a week,” “1–2 times a week,” “2 or 3 times a month,” “Once a month or less frequently,” “I never watch pornography,” and “I never watch pornography intentionally, but others in my surroundings watch it”. The responses were dichotomized into “frequent pornography use” including responses “daily or almost daily” and “3–5 times a week,” and not frequent pornography use including the rest of the response alternatives.

### Statistical Analysis

Since the number of women reporting having bought sex was small (0.4%), the following analyses are restricted to men. Background demographics are presented as proportions by age, educational level and income level using design information and sample weights. Secondly, background demographics with proportions of men having paid for sex are presented by age, educational level, and income level, using design information and sample weights. The crude analysis shows the percentage of men reporting having paid for sex where differences across categories were examined using a chi-square test (*p* < .05). We used multivariate logistic regression to examine the “risk” of having paid for sex in three sequential models. The first model shows the crude estimates, in the second model we controlled for age, educational level and income level. In the subsequent models in addition to Model 2, we added adjustment for the following variables separately, in Model 3 satisfaction with one’s sex life, in Model 4 having sought or found a sex partner online, in Model 5 for having less sex than one would have liked to, and lastly in Model 6 for frequent pornography use. All analyses were carried out using Stata, version 15 (StataCorp).

## Results

In Table 1, background demographics are presented as unweighted and weighed percentages. A total of 9.5% (95% CI: 8.58–10.32) of men reported ever having paid or given other compensation for sex. Men of older age had increased rates of ever having paid for sex. Men with the lowest income level (percentile 1–20) in comparison with the highest income level (percentile 81–100) also showed an increased risk of having paid for sex; however, no significant association was found regarding other income levels. Individuals with 9 years or less of education showed decreased probability of having paid for sex while individuals with 10–12 years of education showed increased probability in comparison with individuals with more than 12 years of education. However, no statistically significant association with educational level remained after adjustment of age and income level.

**Table 1:** Background demographics for men aged 16–84 in Sweden, unweighted and weighted percent, and proportions of men who have paid for sex in percent with 95% CI

Demographics	Men		% paid for sex (CI)
	Unweighted %	Weighted %	
*N*	6048		9.5% (CI 8.58; 10.32)
Age (years)			
16–29	16	23	4.8 (3.23; 6.28)
30–44	21	25	9.9 (7.95; 11.92)
45–64	27	31	11.0 (9.31; 12.72)
65–84	35	21	11.4 (9.86; 12.91)
Educational level			
≤ 9 years	32	21	6.1 (4.5; 7.71)
10–12 years	18	24	11.0 (9.71; 12.35)
> 12 years	50	50	9.0 (7.44; 10.6)
Income level			
0–20	17	18	12.00 (9.57; 14.41)
21–40	20	20	10.1 (8.14; 12.05)
41–60	22	21	9.5 (7.62; 11.42)
61–80	20	20	8.3 (6.41; 10.17)
81–100	21	21	7.6 (5.96; 9.27)

In Table 2, the results of our analysis of the association between sex life characteristics and ever having paid for sex is presented. Men who reported they were dissatisfied (OR: 1.72; 95% CI: 1.34–2.22) had increased probability of ever having paid for sex in comparison with men who were satisfied with their sex life. Furthermore, men who had ever looked for or met sex partners online, were five times more likely to ever having paid for sex (OR: 5.07; 95% CI: 3.97–6.46), compared to men who had not. Men who reported having had less sex than they would have liked to were almost threefold more likely to have paid for sex (OR: 2.78; 95% CI: 2.12–3.66). Likewise, frequent pornography users also had a threefold probability of having paid for sex than other men (OR: 3.02; 95% CI: 2.28–3.98). All sex-life-related variables hence remained statistically significant after adjustment for age, income and educational attainment.

**Table 2:** The odds of having paid for sex by different background and sex life variebls [odds ratios (OR) with confidence intervals (CI) and adjusted odds ratios (aOR)]

Variable	OR	95% CI	aOR^a^	CI	*p* value
Age (years)					<.0001
16–29	1 (REF)		1 (REF)		
30–44	2.21	(1.48–3.31)	2.57	(1.63–4.05)	
45–64	2.48	(1.70–3.63)	2.88	(1.90–4.38)	
65–84	2.58	(1.78–3.73)	2.94	(1.97–4.39)	
Education (years)					<.0001
≤ 9 years	0.66	(0.47–0.92)	0.75	(0.52–1.09)	
10–12 years	1.25	(0.99–1.58)	1.26	(0.99–1.61)	
> 12 years	1 (REF)		1 (REF)		
Income percentile					.1067
1–20	1.65	(1.19–2.30)	1.73	(1.23–2.42)	
21–40	1.36	(0.99–1.88)	1.31	(1.94–1.83)	
41–60	1.28	(0.93–1.76)	1.21	(0.86–1.70)	
61–80	1.10	(0.78–1.55)	1.03	(0.72–1.46)	
81–100	1 (REF)		1 (REF)		
Satisfaction with sex life					.0003
Satisfied	1 (REF)		1 (REF)		
Both satisfied and dissatisfied	1.19	(0.93–1.52)	1.13	(0.87–1.48)	
Dissatisfied	1.66	(1.30–2.14)	1.72	(1.34–2.22)	
Looked for or met sex partner online					<.0001
No	1 (REF)		1 (REF)		
Yes	3.9	(2.80–4.35)	5.07	(3.97–6.46)	
Having less sex than one would have liked					<.0001
No	1 (REF)		1 (REF)		
Yes	2.78	(1.72–2.85)	2.78	(2.12–3.66)	
Frequent porn user					<.0001
No	1 (REF)		1 (REF)		
Yes	1.90	(1.49–2.41)	3.01	(2.28–3.98)	

^a^Adjusted for age, highest educational attainment, and income level

## Discussion

In this study, we took advantage of unique data from the randomized population-based survey SRHR2017, linked with Sweden’s extensive and high-quality nationwide administrative registers, to identify the proportion of men ever having paid or given other types of compensation for sex in Sweden. Our results confirms that the proportion of men reporting ever having paid for sex in our survey (9.5%) is comparable to previous studies and with other Nordic as well as western European countries (Haavio-Mannila & Rotkirch, 2000; Jones et al., 2015; Schei & Stigum, 2010). The age-group with the highest proportion of men having paid for sex was men above the age of 45 years (11%), and men 30–44 years (10%) reported a similar proportion. The lowest proportion was reported among men 16–29 years of age. It is unclear whether this is due to the question, which provides us with a lifetime prevalence that naturally increases with age, or that sex purchase became illegal in Sweden in 1999.

Our results regarding education and income of buyers also confirm previous studies (BRÅ, 2008; Priebe & Svedin, 2011), that buyers are from different socioeconomic backgrounds and educational level is not associated with having paid for sex. However, having a very low income seem to be associated with having paid for sex, which may indicate underlying vulnerability and deprivation. This contradicts the findings of Priebe and Svedin (2011) and Milrod and Monto (2017) that a higher proportion of buyers had high income. This could potentially be due to differences in the participant characteristics since Priebe and Svedin (2011) was based on an online panel which in Sweden usually tends to hold a larger proportion of males, and individuals that are better educated and have higher incomes than the population in general (Bosnjak et al., 2013).

To our knowledge, no study based on a randomized population based survey has explored the relationship between sex life satisfaction and sex purchase, however it does seem reasonable to assume dissatisfaction drives demand, including having less sex than one would have liked to. In our findings, we see a strong association between having looked for or met sex partners online and sex purchase. Our results confirm previous findings that buyers do use internet and/or mobile apps for sexual activity to a higher extent than non-buyers (Monto & Milrod, 2014; Priebe & Svedin, 2011).

Our results show a strong statistically significant association between frequent pornography use and ever having paid for sex. Swedish research has shown that frequent pornography users also have higher levels of risk taking such as alcohol and drug use as well as higher sexual risk taking such as early sex debut and experiences of selling sex, in comparison with non-frequent pornography users (Mattebo, Tydén, Häggström-Nordin, Nilsson, & Larsson, 2013; Svedin, Akerman, & Priebe, 2010).

In all, sex life dissatisfaction and not having as much sex as one would have preferred, as well as online sexual activity and frequent pornography use are strongly associated with having paid for sex among Swedish men. This tells us that these individuals differ from men not having paid for sex in terms of sex life characteristics. It also gives us an indication that they may differ in terms of other factors related to sex life and sexual risk taking but it remains unclear how. Need for intimacy and social dimensions could also play a role (Birch & Braun-Harvey, 2019; Monto & Milrod, 2014). These insights are of importance in the prevention of disease and promotion of sexual health. The understanding of who pays for sex and why is key to reduce demand of sexual services and is of importance not only for law enforcement but also for public health interventions and support activities targeted toward both people paying for and people receiving money or other compensation for sex.

The strengths of this study include the use of the unique data SRHR2017, enriched with high-quality nationwide register data. In prior research, information on sex life factors such as satisfaction, pornography use and online partners is lacking while in our study the results contribute to the understanding of mechanisms driving demand for sex. Some study limitations needs to be taken into consideration in contextualizing the results. First, while the SRHR2017 is a population based sample, the response rate was 31% (i.e., 14,500 participants). Non-response might have biased our results, because many people resist disclosing information about sensitive topics such as sexual activities and experiences of illegal actions. Hence, our outcome measure is likely to be underreported. The outcome measure was “Have you ever paid or given other compensation for sex?” A total of 9.5% of men reported ever having paid for sex, of which 2.8% (of the 9.5%) reported having paid for sex during the past year. However, the question was unfortunately vaguely formulated, where all options was put together in the same question. Hence, we cannot differ between non-response and a selected “no” response. Only 0.26% of all men reported they had purchased sex within the last 12 months, hence we chose not to use this estimate in our analyses. It is unclear as to what extent this may include online purchases since the question did not define online versus offline. Second, the variable of sex life satisfaction referred to the past year, while the rest of our variables measured lifetime prevalence. This is a limitation that sets back our possibility to identify correlations to recent sex purchase. Thirdly, in our study, we have no information on relationship status, which would have helped us further in the understanding of the results.

### Conclusions

Our study provides novel insight on the demand side of sex purchase in the Swedish population. Men in Sweden having paid for sex are from different socioeconomic backgrounds, but are to a higher extent less satisfied with their sex life, report having less sex than they would have wanted to, have experience of online sexual activity and are to a higher extent frequent pornography users in comparison with men who have not paid for sex. These insights needs to be taken into account in support and prevention activities for increased sexual health as well as to end the demand of sexual services.
